# Effects of Oral Gabapentin, Local Bupivacaine and Intravenous Pethidine on Post Tonsillectomy Pain

**Published:** 2015-09

**Authors:** Soroush Amani, Mohamad Reza Abedinzadeh

**Affiliations:** 1 *Department of Otorhinolaryngology, Kashani Hospital, Shahrekod University of Medical Sciences, Shahrekord, Iran.*; 2 *Department of Anesthesiology, Kashani Hospital, Shahrekod University of Medical Sciences, Shahrekord, Iran.*

**Keywords:** Bupivacaine, Gabapentin, Pain, Tonsillectomy

## Abstract

**Introduction:**

Tonsillectomy is one of the most common surgeries performed worldwide. Post-operative pain arising from tonsillectomy is one of the earliest complications that can postpone oral nutrition and increase the hospitalization period. Administration of opioids via injection is usually preferred to relieve pain in these patients. However, the side effects of this approach prompted us to seek alternative treatments. In this study, the effectiveness of oral gabapentin is compared with an intravenous (IV) injection of pethidine and a local injection of bupivacaine in the control of pain after tonsillectomy.

**Materials and Methods:**

This clinical trial was performed on 7-15 year-old patients who were candidates for tonsillectomy at Shahrekord Kashani hospital from 2012–2013. The patients were divided into three groups at random. Group 1 was give 20 mg/kg oral gabapentin 1 hour before anesthesia. In Group 2, 2.5 ml bupivacaine 0.25% was injected into each tonsil bed by a surgeon. In Group 3,1 mg/kg pethidine was injected intravenously after intubation. To assess post-operative pain, the Oucher scale was used in recovery as well as 3,6,12, and 24 hours after surgery.

**Results:**

The pain score was lowest in the gabapentin group and highest in the bupivacaine group during the study. The pain score in the gabapentin group was significantly lower than that in the bupivacaine group (P<0.05). No statistically significant difference was found between the pain score of the Pethidine group and that of the Bupivacaine group (P>0.05).

**Conclusion:**

Gabapentin, with its antihyperalgesic properties and other unknown properties, is a convenient drug for controlling pain following tonsillectomy.

## Introduction

Tonsillectomy is one of the most common childhood surgical procedures, and is performed in over half a million children a year in the US alone (1). Post-operative pain after tonsillectomy, particularly when swallowing, can lead to many complications such insufficient food intake, dehydration, sleep disturbance and increased risk of secondary bleeding (2). Adequate pain control is required to ensure rapid return to oral food intake and hospital discharge; however the analgesics commonly used after tonsillectomy can increase the risk of post-operative complications. Non-steroidal anti-inflammatory drugs increase the risk of post-operative bleeding (3), while opioid medications increase the risk of nausea and vomiting, and also the risk of respiratory depression. Acetaminophen provides less pain relief than opioids, and increases the need for other analgesia (4). 

An effective analgesic that does not increase the risk of post-operative bleeding, nausea, vomiting, or respiratory depression would be extremely useful in the management of tonsillectomy patients (5). Gabapentin may be a medication that fulfils these requirements. Gabapentin is an anti-epileptic drug that probably exerts its effect through selectively interacting with the α2-δ-subunit of voltage-dependent calcium channels (6). Gabapentin has proven its worth in chronic pain conditions, such as diabetic neuropathy, post-herpetic neuralgia and other neuropathic pain states (7–12). Recently, several reports have indicated that gabapentin may have a role in the treatment of post-operative pain (13). Experimental studies have suggested that gabapentin interacts synergistically with naproxen, and results of a recent clinical study in hysterectomy suggest that a gabapentin–rofecoxib combination may be superior to either agent alone for post-operative pain (14). The aim of the present study was to investigate the effect of oral gabapentine in comparison with an iv injection of pethidine and a local injection of bupivacaine in the control of pain after tonsillectomy.

## Materials and Methods

This randomized, double-blind clinical trial was performed in 7–15 year-old patients who were candidates for tonsillectomy at Shahrekord Kashani hospital from 2012–2013. After obtaining permission from the ethics committee of the university and receiving written informed consent, a table of random numbers was used to divide 105 patients into three groups of 35 through a process of computer-generated block randomization. All patients were operated on by one surgeon using the same procedure. Exclusion criteria were fear of surgery (severe restlessness that could influence patient hemodynamics), anxiety, upper respiratory infection, certain comorbidities (heart failure,‍ congenital heart disease, asthma), use of drugs that have an effect on blood pressure or heart rate, and a long duration of surgery (more than 30 minutes) because of bleeding or repeated anesthesia. The method of anesthesia in the three groups was the same. Two minutes before anesthesia, fentanyl (2µg/kg), thiopental Na 5 mg/kg, atracuriom 0.5 mg/kg, and atropine 0.02 mg/kg were given and then patients were intubated and anesthesia was continued with propofol 100 µg/kg/min and O2 (50%) and N2O (50%). As a basic analgesic regimen, all three groups were given acetaminophen 10 mg/kg every 6 hours when oral intake was re-established. Group 1 was given 20 mg/kg oral gabapentin with 50 ml water 1 hour before anesthesia. In Group 2, 2.5 ml bupivacaine 0.25% was injected into the bed of each tonsil by the surgeon (14,15). In Group 3, 1 mg/kg pethidine was injected iv after intubation. To assess post-operative pain, the Oucher scale, a validated and valuable measure for pre-school as well as for primary school-aged children, was used. The Oucher scale consists of six gradations from 0 to 6. Pain was assessed via facial expression. A score of 0 was assigned to a child who was calm and complaisant, and 6 to a child in a state of severe distress, accompanied by constant crying and a grimacing expression (16,17). Pain scores were assessed by the blinded observer anesthetist on arrival, and at 3, 6, 12 and 24 hours after surgery. Descriptive statistics, including the mean and standard deviation, were used for description of continues variables. A chi-square test was used to test the equality of gender across the three groups. An analysis of variance (ANOVA) was used to test the equality of age and weight between the three groups. The ANOVA test was also used to compare the pain at each stage of the study between the three groups. In addition, the parametric repeated measure of analysis of variance with LSD multiple comparison test was applied to analyze the pain scores of the three groups during the study. The data were analyzed using SPSS version 17, and P<0.05 was considered statistically significant.

## Results

A total of 105 patients in three groups participated in this interventional study. The mean age of the patients was 10.06 ± 3.6 years, with no difference between the three groups (P=0.973). Most of the patients were male, but the difference was not statistically significant (P=0.71). The overall weight of the patients ranged from 14 to 80 kg (mean, 32.03 ± 14.1 kg), with no significant difference between the three groups (P=0. 204).

**Fig 1:**
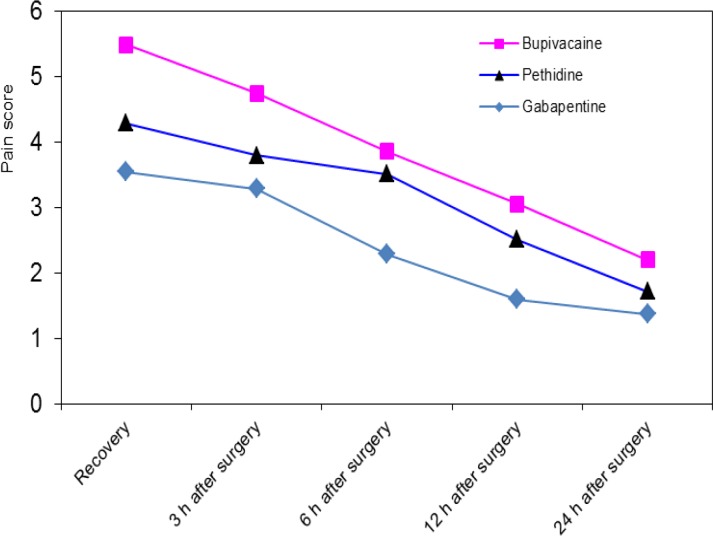
Pain score in he three groups of patients treated with gabapentin, bupivaciane and pethidine at different timepoints following surgery

Age, weight and pain scores in the three groups during the study are shown in (Table 1). In addition, the mean pain score during the study is plotted in Fig 1.

The repeated measure analysis of variance showed a decreasing trend for pain score during the study (Greenhouse-Geisser time factor F-value=52.685; P<0.001) with equal trends for the three groups (Greenhouse-Geisser interaction factor F-value = 0.994; P=0.43) (Fig.1).

**Table 1:** Results of pain score in the three groups of patients treated with gabapentin, bupivaciane and pethidine at different timepoints after surgery

Variable	Gabapentin	Bupivacaine	Pethidine	Test value	P
	Mean ±SD	Mean ±SD	Mean ±SD		
Age	10.2 ± 3.8	10 ± 3.3	10 ± 3.9	0.028	0.973
Weight (Kg)	35.5 ± 15.3	30.4 ± 11.5	30.2 ± 14.8	1.615	0.204
Pain at					
Recovery	3.5 ± 2.5	5.5 ± 3.1	4.3 ± 3.5	3.564	0.032*
3 hour after surgery	3.3 ± 2.7	4.7 ± 2.6	3.8 ± 3	2.508	0.086
6 hour after surgery	2.3 ± 2.1	3.9 ± 2.2	3.5 ± 2.5	4.615	0.012*
12 hour after surgery	1.6 ± 1.3	3.1 ± 2.5	2.5 ± 1.8	5.108	0.008*
24 hour after surgery	1.4 ± 1.2	2.2 ± 1.8	1.71 ± 1.67	2.466	0.090

SD=Standard deviation

* P<0.05 is considered statically significant.

However, there was a significant difference in pain between the three groups (F=4.89; P=0.009). In summary, following the repeated measure analysis of variance, the LSD multiple comparison test showed a significant difference in pain score between the gabapentin and bupivacaine groups during the study (P=0.002). In the gabapentin group, pain severity was less than in the bupivacaine group. Also, there was no significant difference in severity of pain between the pethidine group and the gabapentin group (P=0.11), nor between the pethidine and bupivacaine groups (P=0.133).

## Discussion

Pain management after tonsillectomy is a significant problem, and is usually managed through the use of drugs such as opioids and NSAIDs. Because of the potential complications of these drugs (including nausea, vomiting, and bleeding)and patients’ preference for safe drugs, and because gabapentin is known to relieve pain without causing such complications, this study was conducted to compare the effectiveness of gabapentin with conventional analgesics following tonsillectomy.

Reports from various groups have suggested that gabapentin may play a major role in controlling post-operative pain. For example, Gilron et al. reported that a combination of rofecoxib and gabapentin was superior to either single agent alone in reducing spontaneous and movement pain following abdominal hysterectomy (13). Pandey et al. reported reduced pain and opioid consumption after lumbar discoidectomy and laparoscopic cholecyste- ctomy following pre-operative administer- ation of gabapentin 300 mg (18,19). 

In our study in recovery, there was a significant difference in severity of pain in the three groups 6 and 12 hours after surgery, and there was less pain at all stages in the gabapentin group. In a study performed by Jeon et al. in 2008, 58 adult patients with tonsillectomy were selected and divided in two groups. One group was given placebo and another was given a single dose of gabapentin before surgery. In this study, the gabapentin group needed less analgesic than the placebo group. Pain on swallowing at 2 and 4 hours after surgery was much lower in the gabapentin group, but this difference was no longer seen after this time or during the resting period (20). In our study, pain was reduced in recovery and 6 and 12 hours after surgery in the gabapentin group, but pain during swallowing was not investigated.

In another study performed in 49 patients in Denmark by Mikkelsen et al. in 2006, the first group was given 1,200 mg gabapentin prior to surgery and 600 mg twice on the first day and 600 mg thrice daily on the following 8 days, while the second group was given placebo. The two groups were given 50 mg celecoxib daily, and patients were able to request 2.5 mg morphine. The results revealed that gabapentin decreased the use of opioids in first 24 hours (21). These results are in agreement with our observations concerning the effects of gabapentin.

In a 2006 study by Harleg et al. conducted in the US in patients given gabapentin, the need for opioids and pain after surgery was reduced (22). This result was in agreement with our observations.

In 2008, Nikandish et al. showed that the combination of bupivacaine and pethidine significantly decreased the use of analgesics 4, 6, 8, 12 and 24 hours after surgery, but did not decrease pain during swallowing. They concluded that even though bupivacain and pethidine decreased the use of analgesics, they had an adverse effect on dynamic pain in the 24 hours after surgery (23). Our study also revealed that the use of bupivacaine did not decrease pain in comparison with the pethidine group. In a study performed by Watts et al. (24), there was no significant difference in severity of pain. 

In a 2005 study by Ginstrom et al. in 64 patients, it was shown that use of bupivacaine 5 mg/ml and epinephrine 5 µg/ml was effective in decreasing both bleeding during surgery and the duration of surgery in adult adenotonsillectomy patients (25). We are unable to place our study in the context of this study as we did not assess duration of surgery or amount of bleeding. In another study, Kadar et al. showed that local bupivacaine can decrease pain after surgery (26). This result was not in agreement with our findings, and the difference may be related to the patients’ age and different administration of bupivacaine. For example, use of a local injection allows the drug to be absorbed and metabolize more rapidly compared with packing; allowing a greater and longer-lasting effect on pain. In a 2006 study by Amani et al., 90 patients were divided into three groups and injected with bupivacain, dexamethason or saline (27). As with our results, this study reported no significant difference between the three groups in pain severity after surgery. In a clinical trial reported by Newseland et al. in 2002, 70 patients subjected to tonsillectomy were injected with bupivacaine or normal saline (28). The severity of pain was lowest in the bupivacaine group. This finding is not consistent with our results, and the difference may be related to the dose of drugs used.

The lack of a significant difference in pain relief following gabapentin administration 24 hours post-tonsillectomy may be attributed to the small sample size in the present study. Thus, it is recommended that these three drugs would be compared with respect to pain relief in studies involving a larger sample size.

This study suggests that gabapentin could be an effective drug for managing pain after tonsillectomy, with no notable complications. It is recommended that gabapentin be administered to patients undergoing tonsillectomy.

## Conclusion

According to the results obtained in recovery, and 6 and 12 hours after tonsillectomy, pain severity in the gabapentin group was significantly lower than that in the bupivacain group. With its antihyperalgesic properties and other unknown properties, gabapentin is a convenient drug for controlling pain following tonsillectomy. 
